# Tissue classification for the epidemiological assessment of surgical transmission of sporadic Creutzfeldt-Jakob disease. A proposal on hypothetical risk levels

**DOI:** 10.1186/1471-2458-5-9

**Published:** 2005-01-24

**Authors:** Alberto Rábano, Jesús de Pedro-Cuesta, Kåre Mølbak, Åke Siden, Miguel Calero, Henning Laursen, the EUROSURGYCJD Research Group

**Affiliations:** 1 Laboratory of Neuropathology, Hospital de Alcorcón, Avda Budapest 1 289220 Alcorcón, Madrid, Spain; 2 Applied Epidemiology Department, National Centre of Epidemiology. Carlos III Institute of Health, Sinesio Delgado 6, 28029 Madrid, Spain; 3 Department of Epidemiology, Statens Serum Institut, Artillerivej 5, DK-2300 Copenhagen, Denmark; 4 Neurotec, Division of Neurology. Karolinska Institutet, SE-141 86 Stockholm, Sweden; 5 Department of Spongiform Encephalopathies. National Centre of Microbiology. Carlos III Institute of Public Health, Ctra. Majadahonda-Pozuelo Km. 2,200, 2822 Majadahonda, Spain; 6 Laboratory of Neuropathology, 6301. H:S Rigshospitalet, Blegdamsvej 9, DK-2100 Copenhagen, Denmark

## Abstract

**Background:**

Epidemiological studies on the potential role of surgery in Creutzfeldt-Jakob Disease transmission have disclosed associations with history of specific surgical interventions or reported negative results.

**Methods:**

Within the context of a case-control study designed to address surgical risk of sporadic Creutzfeldt-Jakob Disease in Nordic European countries (EUROSURGYCJD Project), a strategy was adopted to categorise reported surgical procedures in terms of potential risk of Creutzfeldt-Jakob Disease acquisition. We took into account elements of biological plausibility, either clinically or experimentally demonstrated, such as tissue infectivity, PrP expression content or successful route of infection.

**Results:**

We propose a classification of exposed tissues and anatomic structures, drawn up on the basis of their specific putative role as entry site for prion transmission through contact with surgical instruments that are not fully decontaminated.

**Conclusions:**

This classification can serve as a reference, both in our study and in further epidemiological research, for categorisation of surgical procedures in terms of risk level of Creutzfeldt-Jakob Disease acquisition.

## Background

Case-control research on the association between surgery and sporadic Creutzfeldt-Jakob Disease (CJD) encompasses six studies and one meta-analysis [1-8], which frequently yielded diverging, partly inconsistent, positive [5,6] or negative [7,8] results, potentially attributable to methodological difficulties. This paper constitutes the first part of methods development for a case-control study summarily described in the Methods/Design section. "Development" in this sense refers to theoretical development built on scientific evidence and will focus on classification of surgical procedures, using technical and biological criteria appropriate for identifying characteristics of the intervention reflecting the putative risk level of sporadic CJD transmission.

## Methods

In this section, we summarily describe the on-going study, review reports that possibly shed light on the biological plausibility of surgery-related CJD transmission, and propose principles for re-classification of surgical procedures.

### Study design

The on-going case-control study, entitled "Surgery and risk of Creutzfeldt-Jakob Disease" (EUROSURGYCJD), constitutes a Concerted Action funded by the EU Research Commission, contract QLG3-CT-2002-81223. The main objective of this study is to quantify a putative excess risk of CJD associated with surgery. A secondary objective is to establish a basis for the design of preventive strategies. The most relevant methodological characteristics are: case-control design; exposure measurement prior to disease onset, registered as codes for surgical procedures; matched 5:1, randomly chosen population controls and random sample of population controls. The population base is the resident population in Denmark, Finland and Sweden, covered by the respective hospital in-patient registers. Cases are individuals with diagnoses corresponding to ICD-9 codes 046.1 and 331.5 and ICD-10 code A81.0 at death or at hospital discharge for the period 1987–2002, identified from the respective national hospital in-patient registers and corresponding national surveillance units. A questionnaire will be mailed to the heads of the registered hospital department or surveillance unit, and a copy of the medical record will be obtained for diagnosis validation. Approximately 300 patients will fulfil criteria for definite or probable sporadic CJD, and constitute the study cases. Population controls are: 1) 5 × 1 controls (approximately 1500), matched to the corresponding case by age, sex, county of residence of the case (at first discharge from hospital with CJD diagnosis or death if never hospitalised with CJD diagnosis); and 2) a 20/million sample of the 1987–2002 resident population aged >40 years, randomly selected from the corresponding national population registers.

Individual person-numbers will be used for each resident case or control. Diagnoses and surgical procedures at hospital discharge of the corresponding CJD case at any registered time before date of death will be obtained from the three national hospital discharge registers in Sweden, Denmark and Finland. Perusal of surgical records might be undertaken for selected associations in order to understand transmission mechanisms and interpret results. Open-care surgery or dentistry will not be studied. Surgical procedures coded in registers as per national or NOMESCO classifications will be re-classified in accordance with putative levels of transmission risk based on scientific evidence/plausibility.

The quality of CJD diagnoses will be assessed by a review of medical records. The accuracy of surgical history given by the registers will be assessed by comparison with that of a sample of controls and surrogate respondents obtained by interview.

Centralised data analyses will be conducted by the Spanish team. In specific instances, risk due to blood transfusion, whether or not performed during surgical procedures, might also be studied.

### Review of reports and proposal

Surgery may be a pathway for patient-to-patient transmission of sporadic Creutzfeldt-Jakob Disease (CJD). In many invasive surgical procedures, non-disposable surgical instruments come in contact with tissues that are known to be infective in CJD patients. These same instruments may retain a considerable level of infectivity after routine sterilisation, and in successive patients can come into contact with tissues that may act as entry sites for CJD transmission. Among the almost 300 recorded cases of iatrogenic transmission of CJD, 5 cases have been attributed to surgical instruments employed in neurosurgical procedures, whilst 2 additional cases were caused by the use of a contaminated intracerebral EEG electrode [9]. To date, proven surgical transmission of CJD has only been shown to have taken place through instruments contaminated with high-infectivity tissues (brain). However, stainless steel instruments exposed to infective tissue can acquire a maximum load of infectivity in a considerably short period of time (5 minutes) and are highly efficient in transmitting disease even after thorough washing [10]. The possibility of prion transmission through surgical interventions involving nervous or peripheral tissue has raised concern about decontaminating procedures, particularly after the emergence of variant CJD in the United Kingdom and several other countries [11].

In the above-mentioned studies [1-7], surgical procedures have been grouped and analysed according to gross anatomical regions (e.g., thyroid, gallbladder, prostate, etc.), which limits an interpretation of results based on biological inference. For a surgical instrument to act as a vehicle of prion transmission, it should come into contact with infective tissue during surgery of the "donor" (contaminating procedure), should maintain any adhered infectivity after being washed and sterilised, and, finally, should make contact with receptive tissues in the "recipient" patient (transmitting procedure). Different surgical interventions on the same organ may result in direct exposure of different tissues to surgical instruments, and may consequently involve a different risk of prion transmission. Within the context of a case-control study designed to address surgical risk in sporadic CJD in Nordic European countries (EUROSURGYCJD Project), we adopted the strategy of categorising all reported surgical procedures (putative transmitting procedures) in terms of potential risk of CJD acquisition. For this purpose, a classification of exposed tissues and anatomic structures has been drawn up on the basis of their specific putative role as entry site for prion transmission through surgical instruments. This classification can serve, both in our study and in further epidemiological studies, as a reference for a categorisation of surgical procedures in terms of risk of CJD acquisition.

According to the "protein only" hypothesis [12], pathogenic prion protein (PrP^Sc^) is a conformational isoform of PrP^C^, a normal host protein present in neurons and other cell types. In sporadic and familial transmissible spongiform encephalopathies (TSEs), "spontaneous" conversion of PrP^C ^into PrP^Sc ^is the key pathogenic event, followed by the accumulation, deposition and further conversion of PrP^Sc ^in tissues, together with its propagation along specific neural pathways. In the case of transmitted TSEs -when these are not due to direct inoculation into the CNS- a peripheral phase of neuroinvasion by PrP^Sc ^is followed by a subsequent phase of prion replication and propagation along the peripheral nervous system, with final access to the central nervous system [13]. In scrapie, bovine spongiform encephalopathy (BSE) and variant CJD, neuroinvasion follows widespread deposition of PrP^Sc ^in mucosae-associated lymphoid tissue. For the purpose of classifying tissues susceptible to prion inoculation, the following considerations can be derived from this pathogenic model: i) a tissue can act as entry site for prion transmission if it normally expresses PrP^C^; ii) the level of PrP^Sc ^expression of an infected tissue correlates positively with the risk of prion acquisition by that tissue; and, iii) all tissues involved in the propagation chain of infection from peripheral tissues to the central nervous system can act as entry sites for prion transmission.

The recently published WHO classification of tissue infectivity in TSEs [14], though aimed at public health issues radically different from those addressed in our study, may nonetheless serve as a conceptual framework for a tissue classification in terms of risk level of prion acquisition. This approach is based on our above-mentioned assumption (ii). The WHO classification groups tissues in three levels (high, lower and no detected infectivity) on the basis of bioassay infectivity data and/or detection of PrP^Sc ^by Western blot. This three-level classification correlates quite closely with the distribution and levels of PrP^C ^expression in normal nervous and non-nervous tissues in mammals.[15] The WHO tissue classification presents data on vCJD, other human TSEs, BSE and scrapie. Since no vCJD cases have been registered in Nordic countries, our epidemiological study must be limited to sporadic CJD. Consequently, our working classification excludes all tissues where positive data on infectivity or PrP^Sc ^detection have been obtained exclusively in animal TSEs and/or vCJD. This is the case of the small bowel, large bowel (including enteric nerve plexuses), adrenal tissue, pancreas and bone marrow.

Further relevant data for tissue classification derive from iatrogenic CJD cases and from experimental transmission of prion diseases to animals. In roughly half of iCJD cases the entry site has been the CNS or the eye (dura mater transplants, neurosurgical instruments or devices, corneal transplants), whilst in the other half, injection of pituitary hormones means that a peripheral route of entry has to be assumed [9]. Experimental efficiency of prion disease transmission to animals depends on various factors, such as the inoculum dose, the species barrier between the species of origin of the inoculum and the host, and the route of administration, among others. Under the same experimental conditions, different routes of administration show different efficacy of disease transmission, in terms of length of incubation period and percentage of infected animals [16,17]. While the most efficient route of transmission is intracerebral administration, other routes, such as intraperitoneal, intraneural, intraocular, intravenous, subcutaneous and intramuscular administration, have been used successfully in bioassays and other experimental models [18]. Still other routes, such as oral administration and conjunctival instillation [19], have shown a lower efficiency of transmission. Accordingly, clinical and experimental evidence includes several routes of prion transmission that cannot be easily reduced to a simple tissue classification involving tissues of known infectivity in CJD and/or expression of PrP^C ^under normal conditions. This is the case of anterior ophthalmic tissues, skeletal muscle, peritoneum, and subcutaneous tissue rich in sensitive nerve fibres. These anatomical structures have therefore been independently added to our classification as putative routes of entry, with a lower level of risk compared to the high level represented by the central nervous system, sensitive ganglia and posterior eye tissues. The fact that PrP^Sc ^has been recently found in 1/4 skeletal muscle samples of sCJD cases[20] prompted us to classify it as a tissue for potential entry rather than a route.

A final classification of entry sites for putative surgical transmission of CJD contains tissues, including all those showing positive results for sporadic and familial CJD in the WHO classification [14], with minor additions (tonsil and thymus), and maintains the three risk levels of the original classification along with several putative routes of entry, based on clinical and experimental evidence (see Table 1).

**Table 1:** Proposed classification of entry sites for putative surgical transmission of CJD by risk level.

**Risk level**	**Tissues**	**Anatomical structures / routes**
**High**	Brain Spinal cord Retina, optic nerve Spinal ganglia Trigeminal ganglia Pituitary gland Dura mater^a^	

**Lower**	Peripheral nerves^b^ Spleen Lymph nodes^c^ Tonsil^d^ Thymus^d^ Placenta Lung Liver Kidney Blood vessels^e^ Olfactory mucosa CSF Skeletal muscle	Anterior ophthalmic Peritoneum Subcutaneous (high density of sensitive nerve terminals)^f^
**Lowest**	Other	Other

^a^Dura mater does not contain pathological PrP in CJD patients and its infectivity has not been tested. It is included among high-infectivity tissues in the WHO classification because of evidence of iatrogenic transmission through dura mater grafts.^11^The same rationale has been applied to its inclusion in the present table.

^b^Only surgical procedures on peripheral nerves (e.g., amputation, vagotomy, etc.) will be classified according to this tissue.

^c^Surgical procedures that include this tissue as putative risk are those involving direct manipulation of lymph node chains, e.g., lymph node excision, oncological lymphadenectomy, and intra-abdominal procedures with extensive section of lymph node chains, such as cholecystectomy, gastrectomy and diverse types of bowel resection.

^d^Although tonsil and thymic tissue have yielded negative results for infectivity and presence of pathological PrP in sporadic CJD tissue (tonsillar tissue has not yet been tested for infectivity), they are included in the table in the lower level group, together with the spleen and lymph nodes for biological reasons, in order to assess the role of peripheral lymphoid tissue in surgical transmission.

^e^Only procedures involving direct surgery on blood vessels will be classified according to this tissue.

^f^Hand and facial subcutaneous tissue will be included under this heading.

## Discussion

We propose a list intended to be used to generate attributes of single, well-defined surgical procedures and criteria for their classification in terms of putative risk level of transmission. The two main attributes will be: 1) use of non-disposable surgical instruments; and, 2) exposure during surgery of tissues included in the preceding list. High- and lower-risk surgical procedures will be defined by exposure of tissue corresponding to the respective risk level during surgery. In addition, the lowest risk level is represented by those surgical procedures where disposable surgical instruments are not employed or where no listed tissue or anatomical structure is exposed to surgical instruments.

A categorisation of surgical procedures based on the above attributes is inevitably prone to some degree of subjectivity, something that should be minimised by adequate methodological assessment. However, this drawback is more than offset by the possible benefits of identifying specific surgical procedures that pose a significant risk of CJD transmission, in terms of increased study power and control of misclassification of exposure by the removal of surgical procedures, which are probably irrelevant for CJD transmission, from gross anatomical classifications of surgery. We are also aware of the fact that whereas the same surgical instruments are commonly employed in an homogeneous group of procedures in some countries, as is the case of Nordic countries, the same instruments may circulate through a much wider range of procedures and putative risk levels in other countries. Final risk for disease transmission in each surgical procedure combines postulated risk related to tissues exposed in that procedure with the highest risk level of tissues to which instruments have been previously exposed. Accordingly, results should always be interpreted in the light of a known or assumed pattern of instrument circulation between surgical procedure groups. Finally, it is worth stressing that the aim of the approach presented here is exclusively to produce a useful tool for epidemiological research in CJD transmission. Under the present state of knowledge, no consequences for the possible adoption of any practical recommendation concerning surgery or further preventive measures are to be drawn from this approach.

## Competing interests

The author(s) declare that they have no competing interests.

## Authors' contributions

AR assumed the basic task of reviewing literature, proposing tissues and structures, and drafting the first manuscript version. JPC indicated the subject domain, suggested differences between disease transmission and disease acquisition as seen from experimental and observational epidemiological research, generated first paragraphs relating to epidemiological aspects. KM suggested changes in epidemiological aspects. ÅS provided some criticisms. MC gave diverse comments, particularly on biological plausibility. HL clarified the need for methodological refinement in future work. All authors read and accepted the final version.

## Pre-publication history

The pre-publication history for this paper can be accessed here:


